# The endogenous proteoglycan-degrading enzyme ADAMTS-4 promotes functional recovery after spinal cord injury

**DOI:** 10.1186/1742-2094-9-53

**Published:** 2012-03-15

**Authors:** Ryoji Tauchi, Shiro Imagama, Takamitsu Natori, Tomohiro Ohgomori, Akio Muramoto, Ryuichi Shinjo, Yukihiro Matsuyama, Naoki Ishiguro, Kenji Kadomatsu

**Affiliations:** 1 Department of Biochemistry, Nagoya University Graduate School of Medicine, 65 Tsurumai-cho, Showa-ku, Nagoya, Aichi 466-8550, Japan; 2 Department of Orthopaedic Surgery, Nagoya University Graduate School of Medicine, 65 Tsurumai-cho, Showa-ku, Nagoya, Aichi 466-8550, Japan; 3 Department of Orthopaedic Surgery, Hamamatsu University School of Medicine, 1-20-1 Handayama, Higashi-ku, Hamamatsu, Shizuoka 431-3192, Japan

**Keywords:** ADAMTS-4, Spinal cord injury, Extracellular matrix, Chondroitin sulfate proteoglycan, Keratan sulfate proteoglycan, Rat

## Abstract

**Background:**

Chondroitin sulfate proteoglycans are major inhibitory molecules for neural plasticity under both physiological and pathological conditions. The chondroitin sulfate degrading enzyme chondroitinase ABC promotes functional recovery after spinal cord injury, and restores experience-dependent plasticity, such as ocular dominance plasticity and fear erasure plasticity, in adult rodents. These data suggest that the sugar chain in a proteoglycan moiety is essential for the inhibitory activity of proteoglycans. However, the significance of the core protein has not been studied extensively. Furthermore, considering that chondroitinase ABC is derived from bacteria, a mammalian endogenous enzyme which can inactivate the proteoglycans' activity is desirable for clinical use.

**Methods:**

The degradation activity of ADAMTS-4 was estimated for the core proteins of chondroitin sulfate proteoglycans, that is, brevican, neurocan and phosphacan. To evaluate the biological significance of ADMATS-4 activity, an *in vitro *neurite growth assay and an *in vivo *neuronal injury model, spinal cord contusion injury, were employed.

**Results:**

ADAMTS-4 digested proteoglycans, and reversed their inhibition of neurite outgrowth. Local administration of ADAMTS-4 significantly promoted motor function recovery after spinal cord injury. Supporting these findings, the ADAMTS-4-treated spinal cord exhibited enhanced axonal regeneration/sprouting after spinal cord injury.

**Conclusions:**

Our data suggest that the core protein in a proteoglycan moiety is also important for the inhibition of neural plasticity, and provides a potentially safer tool for the treatment of neuronal injuries.

## Background

Following neuronal injury, damaged axons may regenerate or spared axons may sprout, resulting in synaptic reconnection and reconstruction. However, such plasticity after neuronal injury does not occur extensively in the adult mammalian central nervous system (CNS). This is due to a lack of growth-promoting factors, the poor intrinsic regenerative capacity of CNS neurons and the emergence of inhibitory factors after injury [1-7].

Chondroitin sulfate (CS) proteoglycans (CSPGs) are glial scar-associated inhibitors. The inhibitory function of CSPGs on axonal outgrowth is primarily due to their covalently attached CS-glycosaminoglycans. For example, the CS-degrading enzyme chondroitinase ABC (C-ABC) enhances axonal regeneration/sprouting after CNS injury and consequently promotes functional recovery [8,9]. Supporting these data, CSPGs strongly inhibit neurite outgrowth *in vitro*, and C-ABC reverses it [7]. In addition to CSPGs, we found that keratan sulfate proteoglycans (KSPGs) inhibit axonal regeneration/sprouting after injury [10,11]. Thus, *in vivo *neurite outgrowth after a cortical stab wound is enhanced in mice deficient in 5D4-reactive KS in the CNS [10]. These mice also exhibit better motor function recovery after spinal cord injury (SCI) [11]. Furthermore, we found that proteoglycans including both CSPGs and KSPGs inhibit neurite outgrowth *in vitro*, and the KS-degrading enzyme keratanase II reverses this inhibition [11]. These data collectively suggest that glycosaminoglycans (GAGs), such as CS and KS, in a proteoglycan moiety play a central role in proteoglycan-mediated inhibition of neural plasticity after injury.

As both C-ABC and keratanase II are of bacterial origin, repeated use of these enzymes may give rise to adverse effects, such as undesirable generation of antibodies, making their application to therapy limited. Therefore, it would be ideal if we could use an endogenous mammalian enzyme that inactivates the proteoglycans' inhibitory function. However, a fundamental question must be answered before using a mammalian enzyme for this purpose. Namely, since proteoglycan is composed of a core protein and GAGs, it should be determined whether the core protein, in addition to the GAGs, in the proteoglycan moiety is essential for the proteoglycan-mediated inhibition.

Based on the above background, we investigated the effects of a proteoglycan-degrading enzyme, ADAMTS-4 (a disintegrin and metalloproteinase with thrombospondin motifs-4; also designated aggrecanase 1, which stands for an enzyme degrading aggrecan, a common CS/KSPG in the brain and cartilage), on neural plasticity after SCI in this study. As ADAMTS-4 is known to degrade the core protein of some CSPGs, we may determine the importance of the core protein on axonal regeneration/sprouting. Here, we demonstrate that ADAMTS-4 reverses the proteoglycan-mediated inhibition of neurite outgrowth *in vitro*, and promotes functional recovery after SCI.

## Methods

### Spinal cord surgery

Adult female Sprague-Dawley rats weighing 200 to 230 g were used in this study. The animals were anesthetized by intraperitoneal injection of ketamine (100 mg/kg) and xylazine (10 mg/kg). After Th10 laminectomy, we exposed the dura mater and induced injury using a force of 200 kydn with a commercially available spinal cord injury device (Infinite Horizon Impactor; Precision Systems & Instrumentation, Lexington, KY, US). Immediately after the spinal cord contusion, we performed a Th11 partial laminectomy and inserted a thin silicone tube with an osmotic mini-pump into the subarachnoid cavity under a surgical microscope. The osmotic mini-pump (Alzet pump model 2002 (Durect Corporation., Cupertino, CA, US): 200 μl solution, 0.5 μl/hour, 14-d delivery) was filled with recombinant ADAMTS-4 (100 nM) (R&D Systems, Minneapolis, MN, US), C-ABC (0.05 units/200 μl) (Seikagaku Corporation, Tokyo, Japan) or PBS (as a vehicle control). The tube was sutured to the spinous process to anchor it in place and the minipump was placed under the skin on the animal's back. Afterward, the muscles and skin were closed in layers. The bladder was compressed by manual abdominal pressure twice a day until bladder function was restored. Food was provided on the cage floor, and the rats had no difficulty reaching their water bottles. All animals were given antibiotics in their drinking water (1.0 ml Bactramin (Roche, Basel, Switzerland) in 500 ml acidified water) for two weeks after SCI. All animals were treated and cared for in accordance with the Nagoya University Graduate School of Medicine guidelines pertaining to the treatment of experimental animals.

### Western blot analysis

To determine the ADAMTS-4 expression in the injured spinal cord of the rats, the diffusion of His-tagged ADAMTS-4 infused into the injured spinal cord tissue and the degradation of brevican and neurocan by recombinant ADAMTS-4 enzyme, tissue extracts were analyzed by SDS-PAGE/Western blot with anti-ADAMTS-4, anti-His-Tag, anti-brevican, and anti-neurocan antibodies. Samples of the supernatant fraction were collected after centrifuging at 10000 *g *for 15 minutes and were separated by electrophoresis on 5% SDS-PAGE. Proteins were then blotted onto nitrocellulose membranes. Blots were blocked with 5% fat-free dry milk in PBS for 60 minutes and incubated overnight at 4°C with the primary antibody (anti-ADAMTS-4(1000 × dilution; Santa Cruz Biotechnology, Santa Cruz, CA, US), anti-His-Tag (1000 × dilution; MBL, Nagoya, Japan), anti-brevican (1000 × dilution; BD Biosciences, San Jose, CA, US), and anti-neurocan (1000 × dilution; clone 1G2, Seikagaku Corporation) ) in PBS containing 0.3% Triton X-100. They were washed and then were incubated with a second antibody (horseradish peroxidase-conjugated anti-goat and anti-mouse immunoglobulin G (IgG; 5000 × dilution; Jackson ImmunoResearch, West Grove, PA, US)) in PBS containing 0.3% Triton X-100 at room temperature for 60 minutes. Anti-β-actin antibody (1000 × dilution; Sigma, St. Louis, MO, US) was also used as indicated. Bound antibodies were visualized with an ECL and ECL-plus Western blotting detection kit (GE Healthcare, Buckinghamshire, UK).

### Fluorescent assay of ADAMTS-4 activity

To measure ADAMTS-4 activity in the rat spinal cord, we used the SensoLyte^® ^520 Aggrecanase-1 Fluorometric Assay Kit (AnaSpec, Fremont, CA, US). A tissue sample from the rat spinal cord was diluted in 50 μl of assay buffer in a 96-well white plate (Sumitomo Bakelite, Tokyo, Japan). Then, 50 μl of aggrecanase substrate solution was added into each well and incubated for 60 minutes at room temperature. Fluorescence was measured at 30°C using a POWERSCAN 4 (DS Pharma Biomedical, Osaka, Japan) equipped with a 490-nm excitation filter and a 520-nm emission filter.

To evaluate the thermo-stability of ADAMTS-4, the enzyme (10 nM) was incubated *in vitro *at 37°C. After two weeks incubation, the enzyme activity was measured.

### Cell culture

Sprague Dawley rats were killed on postnatal days 7 to 9, and the cerebella were collected. The meninges were carefully removed with fine forceps and the remaining tissues were minced and digested using a Papain Dissociation System (Worthington Biochemical, Lakewood, NJ, US). Dissociated cells were applied to a 35/60% two-step Percoll gradient and centrifuged at 3000 *g *for 15 minutes. Cerebellar granule neurons at the interface were collected. Cells were suspended in neurobasal medium (Invitrogen, Carlsbad, CA, US) supplemented with 2% B27 (Invitrogen), 2 mM glutamine, an additional 20 mM KCl, 50 U/ml penicillin, and 50 μg/ml streptomycin.

Primary cultures of cerebral cortical astrocytes were prepared from newborn Sprague-Dawley rats as previously described [12,13]. Briefly, forebrains were removed aseptically from the skulls, the meninges were excised carefully under a dissecting microscope and the cortices were isolated. The small tissues obtained by mincing the cortices were cultured in flasks in DMEM containing 10% fetal bovine serum (FBS), then incubated at 37°C in a humidified atmosphere containing 5% CO_2_. The culture medium was renewed every three to five days. Experiments were performed on confluent 30-day-old cultures. More than 95% of the obtained cells were glial fibrillary acidic protein (GFAP)-positive. Astrocytes were plated at a density of 5 × 10^5 ^cells per 3.5-cm dish.

Microglia-enriched cultures were obtained using the method of Giulian et al. [14]. Briefly, small pieces of tissues were obtained by mincing the cortices as described above for the astrocyte primary culture and then were cultured in flasks in Mi-medium (DMEM, 10% FBS, 0.2% glucose and insulin 5 μg/ml). The mixed glial culture grown for 21 days was subjected to shaking at 200 rpm on a gyratory shaker for 30 minutes. The detached cells (mainly microglia) were reseeded in fresh culture flasks, and after two hours any contaminating oligodendrocyte progenitors were detached with Tris-buffered saline containing 1 mM ethylenediaminetetraacetic acid (EDTA). This procedure routinely provides a firmly attached homogeneous population of microglia. Microglia were cultured in DMEM containing 10% FBS. More than 95% of the obtained cells were found to be Iba1-positive. Microglial cells were plated at a density of 1 × 10^5 ^cells per 3.5-cm dish.

### RNA extraction and reverse transcription-polymerase chain reaction (RT-PCR)

The total RNA of the cells was isolated using an RNeasy Tissue Mini Kit (Qiagen, Valencia, CA, US) according to the manufacturer's instructions. One microgram of purified total RNA was transcribed using SuperScript III First-Strand Synthesis Super Mix (Invitrogen). The cDNA products were used for the polymerase chain reactions (PCRs), which were performed using a Veriti 96-well Thermal Cycler (Applied Biosystems, Foster City, CA, US) according to the following protocol: 35 cycles of denaturing at 94°C for 30 seconds, annealing at 58°C for 30 seconds, and elongation at 72°C for 60 seconds. The rat ADAMTS-4 primers were 5'-ctacaaccaccgaaccgac-3' (forward) and 5'-tgccagccaccagaactt-3' (reverse). The rat glyceraldehyde-3-phosphate dehydrogenase (GAPDH) primers were 5'-tatgactctacccacggcaag-3' (forward) and 5'-tgcattgctgacaatcttgag-3' (reverse).

### PG degradation assay by ADAMTS-4

Whole brains were isolated from adult female Sprague-Dawley rats. Tissues were homogenized in PBS containing 10 mM *N*-ethylmaleimide and protease inhibitor mixtures (Nacalai Tesque, Kyoto, Japan) using a Dounce-type homogenizer. Homogenates were centrifuged at 24000 *g *for 30 minutes and supernatants were applied to diethylaminoethyl (DEAE) Sepharose (GE Healthcare). Samples were washed three times with wash buffer (50 mM Tris-HCl, pH 7.5, 2 M urea, 0.25 M NaCl, 20 mM EDTA, 0.2 mM PMSF, 1 mM *N*-ethylmaleimide), and the proteoglycans were eluted with 2 M NaCl. The eluent was concentrated using a size-exclusion spin column (molecular weight cutoff, 100 kDa), and the protein concentration was determined using a Micro BCA Protein Assay kit (Thermo Fisher Scientific, Waltham, MA, US). Purified PG samples were incubated with 1 μM recombinant human ADAMTS-4 and ADAMTS-13 (R&D Systems) (diluted in 10 mM Tris-HCl, 0.15 M NaCl, and 10 mM CaCl_2_) for 3 hours at 37°C. Controls included samples with ADAMTS-4 that were inactivated by heating at 95°C for 30 minutes. After a 3 hour incubation period, 1 M dithiothreitol (DTT) SDS-PAGE sample buffer was added to the samples, and then the samples were heated at 95°C for 5 minutes and subjected to SDS-PAGE and Western blotting. Membranes were probed with mouse anti-brevican, anti-neurocan and anti-phosphacan (1000 × dilution), and the primary antibody was detected with anti-mouse, anti-rabbit IgG conjugated to horseradish peroxidase. Bound antibodies were visualized with an ECL Western blotting detection kit.

### Neurite outgrowth assays

Sprague Dawley rats were killed on postnatal days 7 to 9, and the cerebella were collected. The meninges were carefully removed with fine forceps and the remaining tissues were minced and digested using a Papain Dissociation System. Dissociated cells were applied to a 35/60% two-step Percoll gradient and centrifuged at 3000 *g *for 15 minutes. Cerebellar granule neurons at the interface were collected. Cells were suspended in neurobasal medium supplemented with 2% B27, 2 mM glutamine, an additional 20 mM KCl, 50 U/ml penicillin, and 50 μg/ml streptomycin. Four-well chamber slides (NUNC, Roskilde, Denmark) were coated with 20 μg/ml poly-L-lysine (PLL; Sigma) and left overnight at 4°C and then were coated with chick brain proteoglycans (Millipore Bioscience Research Reagents, Temecula, CA, US) and left for 4 hours at 37°C. If indicated, proteoglycans were treated with 10 nM ADAMTS-4 or 200 mU/ml C-ABC in PBS at 37°C. Cerebellar granule neurons were seeded onto four-well chamber slides at 2.0 × 10^5 ^per well. Twenty-four hours after seeding, the neurons were fixed with 4% paraformaldehyde/PBS and stained with anti-neuron-specific β-tubulin (Covance, Princeton, NJ, US) to visualize neurites. Neurite lengths were measured from at least 100 neurons that had neurites longer than twice the cell body diameter, per condition from duplicate wells, and quantified as described previously [15]. The number of adherent cells was counted under 200 × magnification (six fields).

### Behavioral test

The locomotor performance of animals was analyzed using the Basso, Beattie and Bresnahan scale (BBB) open-field score for eight weeks, since the BBB has been shown to be a valid locomotor rating scale for rats. The evaluations were made by two blind observers for all analyzed groups. Briefly, the BBB is a twenty-one-point scale that provides a gross indication of locomotor ability and determines the phases of locomotor recovery and features of locomotion. The BBB score was determined for nine rats in each group.

### Immunohistochemistry

Rats were perfused transcardially under deep ether anesthesia with buffered 4% paraformaldehyde. The spinal cords were removed, postfixed in 4% paraformaldehyde overnight and cryoprotected in buffered 30% sucrose during the subsequent night. Tissues were cut into 20 μm sections with a cryostat and mounted on glass slides. Sections were blocked in PBS containing 3% BSA. Sections were then incubated with 5-hydroxytryptamine (5-HT) antibody (100 × dilution; Immunostar, Hudson, WI, US) in a blocking solution overnight at 4°C in PBS containing 3% BSA. After rinsing in PBS, the sections were incubated with the secondary antibody (100 × dilution; Alexa 488 conjugated anti-rabbit antibody; Invitrogen) for 60 minutes at room temperature. Subsequently, the sections were rinsed in PBS, mounted with FluorSave (Calbiochem, San Diego, CA), and examined by an Olympus model BX41 microscope fitted with the appropriate filters.

### Morphometry

The epicenter of a lesion was determined by hematoxylin and eosin staining of several of the serial 20 μm sections. All the cross-sectional image analyses were performed using spinal cord samples from positions 5 mm caudal to the lesion site. Mean values for each animal were then compared. Light intensity and thresholding values were maintained at constant levels for all analyses by a computer-driven microscope stage (MetaMorph Offline version 6.3 *r*^2^; Molecular Devices, Sunnyvale, CA, US). The amounts of axonal outgrowth of the wound area were assessed by counting signals visualized by staining with anti-5-HT antibody, for 650 × 860 μm^2 ^counting frames around a lesion. Statistical analyses were performed for five rats for each experimental group.

### Statistical analysis

Using SPSS (SPSS Inc., Chicago, IL, US), data for the BBB score were analyzed by repeated measures analysis of variance (ANOVA) with a post hoc Bonferroni test. Data were statistically analyzed using an unpaired two-tailed Student's *t*-test for histological assessment for the area of 5-HT-positive fiber. In all statistical analyses, values of *P *< 0.05 were considered to indicate significance. When gathering data for the statistical analyses, the investigators were blinded to each group in all procedures.

## Results

### ADAMTS-4 expression and activity in the injured spinal cord of rats

**Figure 1:**
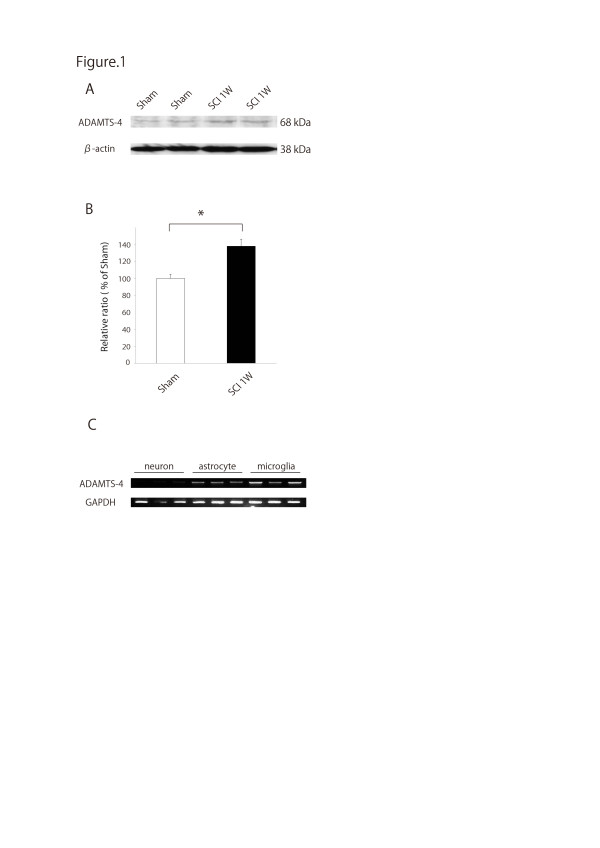
**ADAMTS-4 expression and activity in the injured spinal cord of rats**. ADAMTS-4 in the spinal cord tissue lysates was slightly increased after spinal cord injury compared with the sham-operated rats (Western blot analysis) (**A**). The proteolytic activity of ADAMTS-4 was significantly increased by about 1.4-fold in the rat spinal cord after injury (**B**). Enzymatic activities of tissue extracts of the spinal cord (1 cm around the lesion) were measured. *, *P *< 0.01. ADAMTS-4 mRNA was mainly expressed by astrocytes and microglia, but not neurons (**C**).

The level of ADAMTS-4 protein was slightly increased after SCI, as revealed by Western blot analysis of the spinal cord lysate seven days after injury (Figure 1A). ADAMTS-4 is known as a degrading enzyme for the CS/KSPG aggrecan. To determine whether its expression after SCI was accompanied by an elevation of proteolytic activity, an assay using fluorescent substrate was employed. The spinal cord lysate seven days after injury showed a significant elevation of the aggrecan-degradation activity of ADAMTS-4 (approximately 1.4 fold versus sham-operated rats) (Figure 1B).

### Astrocytes and microglia express ADAMTS-4

To determine which cells express ADAMTS-4 in the CNS, we performed an RT-PCR analysis. Strong ADAMTS-4 expression was detected in primary cultured astrocytes and microglia, but not in neurons (Figure 1C).

### ADAMTS-4 degrades the endogenous CSPGs brevican, neurocan and phosphacan

**Figure 2:**
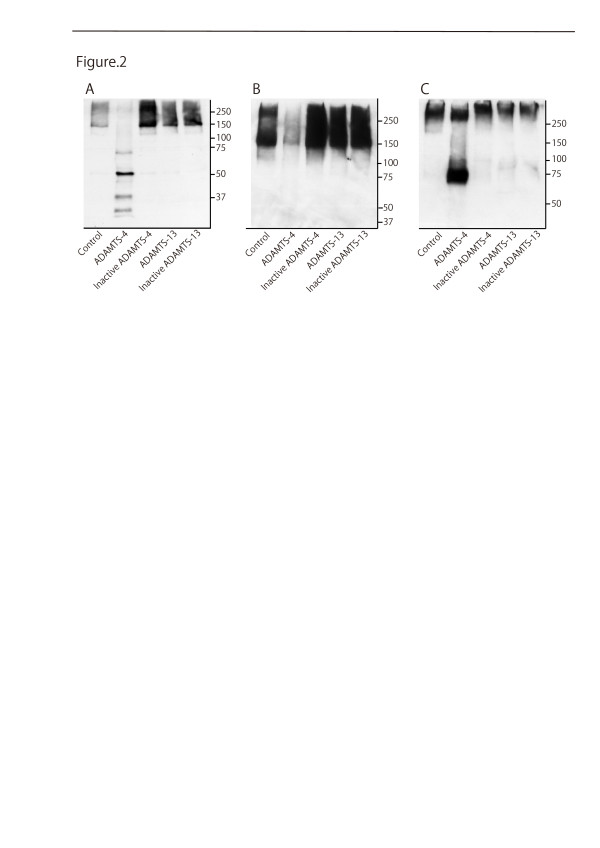
**Cleavage of CSPGs by ADAMTS-4**. Cleavage of brevican (**A**), neurocan (**B**), and phosphacan (**C**) by human recombinant ADAMTS-4. CSPGs were incubated with the indicated enzymes and their degradation was estimated by Western blot analysis as described under the **Methods**. ADAMTS-4 degrades brevican, neurocan and phosphacan, whereas inactive ADAMTS-4, which had been heat-denatured, and ADAMTS-13 do not degrade these CSPGs. CSPGs, chondroitin sulfate proteoglycans.

To explore the possibility that ADAMTS-4 has a wide range of substrates, not limited to aggrecan, we further examined its degradation activity on endogenous CSPGs in the CNS. To this end, homogenates of rat brain were applied to a DEAE column, and highly negatively-charged species were eluted with 1 M NaCl. When tissue extracts of the spinal cord were directly used for Western blotting, many degradation products of proteoglycans were detected even without SCI (data not shown). Therefore, to estimate the degradation activity of ADAMTS-4, a gross purification of PGs with a DEAE column was needed. Indeed this purification provided PGs with over 100 kDa (Figure 2A-C, Control). The grossly purified proteoglycans were incubated with active or heat-inactivated human recombinant ADAMTS-4 or ADAMTS-13 for 3 hours. ADAMTS-13 has a thrombospondin type I sequence repeat motif and peptidase activity as ADAMTS-4 does, but shows distinct substrate specificity, that is, it specifically digests von Willebrand factor and regulates blood coagulation. Furthermore, we found that ADAMTS-13 expression was upregulated after SCI of rats (Tauchi et al., unpublished data). Therefore, we used ADAMTS-13 as a negative control. After incubation, the samples were subjected to Western blot analysis to detect the levels of brevican, neurocan and phosphacan. Incubation with ADAMTS-4 resulted in cleavage of brevican (Figure 2A, ADAMTS-4), which is consistent with a previous report [16,17]. We also demonstrated that ADAMTS-4 degraded neurocan and phosphacan (Figure 2B, C, ADAMTS-4). Proteolytic activity was attenuated when ADAMTS-4 was heat-inactivated prior to incubation with substrate (Figure 2A-C, Inactive ADAMTS-4). In contrast, ADAMTS-13 as well as its heat-inactivated form did not degrade these proteoglycans (Figure 2A-C, ADAMTS-13 and Inactive ADAMTS-13). Consistent with our data, there has been no report thus far that ADAMTS-13 cleaves proteoglycans, although it effectively cleaves von Willebrand factor [18].

### ADAMTS-4 reverses proteoglycans' inhibition of neurite growth

**Figure 3:**
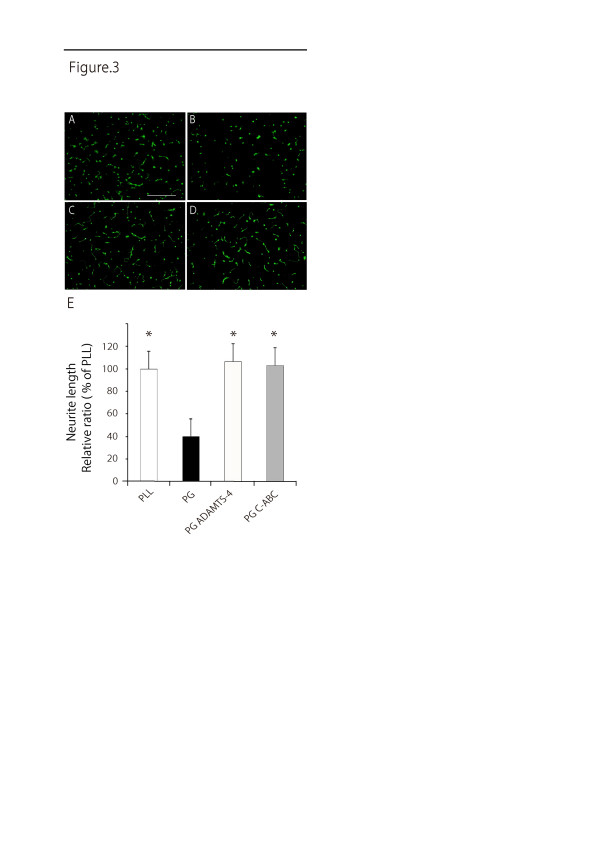
**ADAMTS-4 restores neurite outgrowth**. P8 rat cerebellar granular neurons were cultured on PLL (**A**) or PG extracted from chick brains (**B**). ADAMTS-4 (**C**) and C-ABC (**D**) treatments restored the neurite outgrowth. Scale bar, 200 μm. (**E**) The quantification of **A **to **D**. Data represent the relative ratio of the average neurite length ± SD comparing PLL. *, *P *< 0.01 (versus PG: repeated-measures ANOVA). ANOVA, analysis of variance; C-ABC, chondroitinase ABC; PG, proteoglycan; PLL, poly-L-lysine.

We next examined the effects of ADAMTS-4 on proteoglycan-mediated inhibition of neurite outgrowth. To this end, cerebellar granule neurons were primary cultured on PLL or chick brain-derived PGs coated on PLL on a tissue culture dish. PGs coated on the substratum strikingly inhibited neurite outgrowth of primary neurons as compared with the PLL control (Figure 3A, B, E). Notably, ADAMTS-4 blocked the PG-mediated inhibition and this blocking effect was comparable to that of C-ABC (Figure 3C-E).

### ADAMTS-4 promotes motor function recovery after SCI

**Figure 4:**
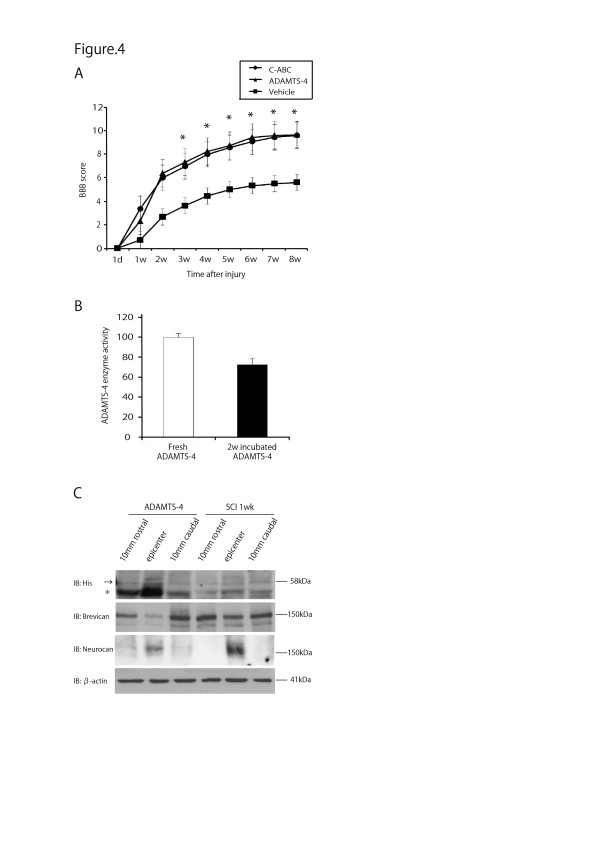
**BBB score and the diffusion of exogenous ADAMTS-4 after SCI**. The graph of BBB score shows data from nine rats for each group at each time point (**A**). Values are presented as the means ± SEM. *, *P *< 0.05 (versus vehicle: repeated-measures ANOVA). ADAMTS-4 retained enzymatic activity after two weeks incubation at 37°C (**B**). Brevican and neurocan were regionally degraded by ADAMTS-4 around the infused area of the spinal cord (**C**). His-tagged recombinant ADAMTS-4 (58 kDa: arrow) was detected at the epicenter of the injured spinal cord of the ADAMTS-4 treated rat. ANOVA, analysis of variance; BBB, Basso, Beattie, and Bresnahan; d, day; SCI, spinal cord injury; SEM, standard error of the mean; w, week.

The data so far shown indicated that ADAMTS-4 degraded CSPGs, which are known to inhibit neuronal axon regeneration after injury. However, the level of endogenous ADAMTS-4, the expression of which is enhanced after SCI, appeared to be insufficient to overcome the inhibitory activity of CSPGs, because SCI leads to serious functional disturbance. Therefore, we next investigated whether exogenous ADAMTS-4 would ameliorate SCI. The adult rat spinal cord was inflicted with a force of 200 kdyn at the Th10 level. ADAMTS-4 or C-ABC was locally infused at the injury site using an osmotic pump for two weeks. The motor function recovery was then evaluated using the BBB score. As shown in Figure 4A, ADAMTS-4 and C-ABC significantly improved motor function after SCI, compared to the vehicle group (*P *< 0.05, respectively: repeated measure ANOVA). Notably, the effects of ADAMTS-4 and C-ABC were comparable (Figure 4A). Therefore, our data demonstrated that exogenous ADAMTS-4 promoted motor function recovery after SCI.

To evaluate the thermo-stability of ADAMTS-4, we incubated ADAMTS-4 solution (10 nM) *in vitro *for two weeks at 37°C. It retained about 70% activity of the fresh ADAMTS-4 (Figure 4B). Furthermore, we examined the extent of ADAMTS-4 diffusion into the tissue after osmotic pump infusion. As the infused ADAMTS-4 was tagged with His, its molecular weight was 58 kDa. The 58 kDa band was detected at the epicenter of ADAMTS-4 treated rat on Western blot analysis (Figure 4C, arrow). A band slightly lower than 58 kDa also appeared most strongly at the epicenter in ADAMTS-4 treated rats (Figure 4C, asterisk), suggesting that it might represent a processed form of the infused ADAMTS-4 although its enzymatic activity was uncertain. In accordance with this result, brevican and neurocan were degraded at the injury epicenter of ADAMTS-4 treated rat (Figure 4C). These data collectively suggest that exogenous ADAMTS-4 cleaved its substrates around the infused area and, consequently, contributed to the functional recovery after SCI.

### ADAMTS-4 promotes axonal regeneration/sprouting

**Figure 5:**
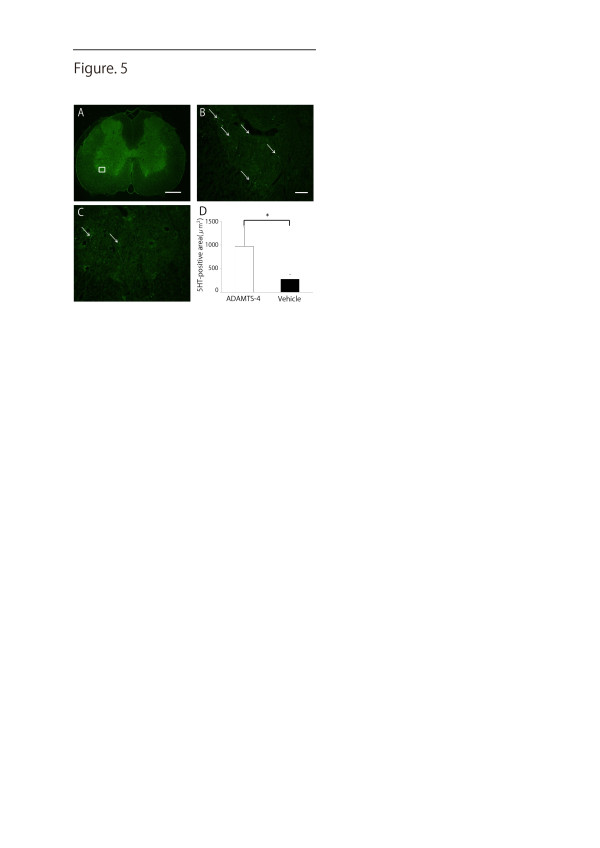
**Immunofluorescence analysis after SCI**. 5-HT staining of the ventral horn (**A-C**, 5 mm distal to the lesion) is shown for the ADAMTS-4 treatment group (**A **and **B**) and vehicle group (**C**). Higher-magnification of the boxed area in **A **is shown in **B**. The arrows in **B **and **C **indicate 5-HT-positive fibers. Scale bars, 500 μm (**A**) and 50 μm (**B **and **C**). (**D) **The 5-HT-positive areas are summarized in the graph. Five rats are used for each group at eight weeks after spinal cord injury. *, *P *< 0.05. Values are presented as the means ± SD. 5-HT, 5-hydroxytryptamine; SCI, spinal cord injury.

Regeneration/sprouting of the serotonergic descending raphespinal tract may partly explain the reason for motor function recovery after SCI in rodents [19]. We stained tissues 5 mm distal to the lesion for 5-HT, since serotonergic axons are 5-HT-positive. 5-HT-positive fibers were more abundantly found in the ventral horn of the gray matter in the ADAMTS-4-treated rats than in the vehicle rats (5-HT-positive area: ADAMTS-4 treated rats, 971 ± 407 versus the vehicle rats, 278 ± 102; *P *< 0.05) (Figure 5A-D).

## Discussion

### Is the degradation of ECM components beneficial or detrimental to the process of neuronal injuries?

The extracellular matrix (ECM) maintains the integrity of the CNS both structurally and functionally. Matrix metalloproteinases (MMPs) are upregulated after cerebral infarction and are implicated in stroke pathology [20,21]. Indeed, an MMP inhibitor has been shown to reduce infarct size [22]. MMPs facilitate degradation of ECM proteins, including collagen and laminin, loosen the blood-brain barrier and promote the infiltration of inflammatory cells. In addition, ADAM-17 enhances shedding of tumor necrosis factor (TNF) after ischemia [23] and an ADAM-17 antagonist reduces infarct size after transient middle cerebral artery occlusion [24]. Taken together, these results indicate that ECM-degrading enzymes, that is, MMPs and ADAM-17, worsen the functional disturbance after stroke by promoting deconstruction of the blood-brain barrier and infiltration of the inflammatory cells. In this context, an unsolved question is whether ADAMTS-4 is beneficial or detrimental to the process of neuronal injuries, because CSPGs are the major component of the CNS ECM, and ADAMTS-4 is the most potent enzyme to degrade core proteins of CSPGs, which include brevican, aggrecan, versican, neurocan and phosphacan [16,17,25-27]. This report shows for the first time that ADAMTS-4 is beneficial to the process of a neuronal injury, namely, SCI.

### ADAMTS-4 as a potent therapeutic agent for neuronal injuries

A characteristic of the CNS ECM is that it is rich in CSPGs and hyaluronan [28]. CSPGs may play a role in the maintenance of the correct hydrodynamics through their negatively charged properties, but also may contribute to the maintenance of neuronal networks by inhibiting synaptic plasticity [29-31]. Among CSPGs, the lectican family members (brevican, aggrecan, versican and neurocan) bind hyaluronan through their globular N-terminus [32]. This aggregated complex of matrix composes perineuronal nets, confers stability in neuronal networks, and inhibits plasticity after injuries [33]. ADAMTS-1, -4, -5, -8 and -9 degrade aggrecan, the most abundant CSPG in the cartilage and are implicated in cartilage degradation [25,26,34]. ADAMTS-4 degrades aggrecan, versican and brevican [16,17,25-27]. In addition to these findings, we showed here that neurocan and phosphacan were also cleaved by ADAMTS-4. We also found that ADAMTS-4 was upregulated in the spinal cord after SCI. Finally, we demonstrated that exogenous ADAMTS-4 promoted functional recovery after SCI. Notably, the effects of ADAMTS-4 and C-ABC on functional recovery were comparable. Regarding the function of exogenous ADAMTS-4, we cannot exclude the possibility that exogenous ADAMTS-4 exerts neuroprotection. However, we observed that ADAMTS-4 promoted neurite outgrowth *in vitro *and axonal regeneration/sprouting *in vivo*. Considering also an increasing body of evidence that CSPGs work as a potent inhibitor for neural plasticity as mentioned above, it is most likely that exogenous ADAMTS-4 essentially promoted axonal regeneration/sprouting and consequently neuronal network reconstruction. Unlike the expressions of C-ABC and keratanase II, ADAMTS-4 expression is found in mammals and is detected in the spinal cord. Furthermore, human ADAMTS-4 is available. Therefore, this enzyme could be used for humans without concerns about adverse effects, such as the generation of antibodies, and is a candidate therapeutic agent for neuronal injuries.

### The importance of the core protein in the proteoglycan-mediated inhibition of neural plasticity

The ADAMTSs are a group of proteases that are found both in mammals and invertebrates. The ADAMTSs are extracellular, multidomain enzymes whose known functions include: 1) collagen processing, 2) cleavage of the matrix proteoglycans, 3) inhibition of angiogenesis, and 4) blood coagulation homoeostasis [35]. It is known that ADAMTS-1, -4 and -5 cleave substrates at specific Glu-X bonds [27]. Thus, the target of ADAMTS-4 is the core protein in a proteoglycan moiety. We also targeted the core protein by heat-inactivating or reducing/alkylating and found that those proteoglycans lost their inhibitory activity on neurite growth [36]. Taken together, our data highlight the importance of the core protein in the proteoglycan-mediated inhibition of neural plasticity.

### The biological significance of the upregulation of ADAMTS-4

It has been reported that CSPGs, including brevican, neurocan, phosphacan, versican and NG2, are upregulated after CNS injuries [37-42]. These CSPGs are also upregulated after SCI [43]. We demonstrated here that ADAMTS-4 expression was also increased following SCI. An increase in ADAMTS-4 expression has also been reported in the ipsilateral cerebral hemisphere following transient middle cerebral artery occlusion in the rat [44], suggesting that the upregulation of ADAMTS-4 may be a general phenomenon induced by neuronal injuries.

We demonstrated that ADAMTS-4 could degrade brevican, neurocan and phosphacan, and reversed the neurite growth inhibition mediated by chick brain proteoglycans. Moreover, local administration of ADAMTS-4 promoted axonal regeneration/sprouting and motor function recovery after SCI. On the other hand, this finding also indicates that the level of endogenous expression of ADAMTS-4 is not sufficient to confer plasticity and functional recovery after SCI. The cleavage of CSPGs induced by neuronal injuries may be achieved not only by ADAMTS-4 but also by MMPs, which are also upregulated after neuronal injuries [20,21,45]. However, MMPs also cleave other matrix proteins, such as collagen and laminin, loosen the blood-brain barrier, enhance the infiltration of inflammatory cells, and worsen pathology. An elevation of inflammatory cytokines, for example, TNF-α, in turn enhances ADAMTS-4 expression [46]. Therefore, a complex molecular and cellular network may be established after neuronal injuries to regulate the balance between the production and removal of inhibitory factors for neural plasticity.

In this context, it is of note that the expressions of ADAMTS-1 and -4 are elevated in rats with kainite induced CNS lesions [47]. ADAMTS-4, in particular, is strikingly upregulated and its well-known substrate brevican is cleaved where ADAMTS-4 is expressed. Thus, the report by Yuan et al. clearly demonstrates that ADAMTS-4 expressed *in vivo *is actually proteolytically active *in situ *in the CNS. Our study also demonstrated that heat-inactivated ADAMTS-4 lost its activity promoting neurite outgrowth against proteoglycans. Taken together, these data support the idea that the proteolytic activity of ADAMTS-4 is central for its contribution to functional recovery after SCI. On the other hand, it has also been reported that a proteolytically inactive point mutant of ADAMTS-4 promotes neurite outgrowth extension through the activation of ERK kinase [48]. Thus, this interesting phenomenon should also be taken into consideration as a potential mechanism.

## Competing interests

The authors declare that they have no competing interests.

## Authors' contributions

RT, SI, and RS performed *in vivo *experiments. RT, TN, TO and AM carried out *in vitro *experiments. RT, SI, YM, NI and KK designed the study. RT and KK wrote the manuscript. All authors read and proved the final manuscript.
